# “Outcome of non-surgical periodontal treatment on Gal-1 and Gal-3 GCF levels in periodontitis patients: a case-control study”

**DOI:** 10.1007/s00784-024-05688-1

**Published:** 2024-05-14

**Authors:** Nayroz Abdel Fattah Tarrad, Olfat Gamil Shaker, Riham Mohamed Hassan Elbanna, Maha AbdelKawy

**Affiliations:** 1 Oral Medicine and Periodontology Department, Faculty of Dentistry, Fayoum University, Fayoum, Egypt; 2 Medical Biochemistry and Molecular Biology Department, Faculty of Medicine, Cairo University, Cairo, Egypt; 3 Oral Medicine and Periodontology Department, Faculty of Dentistry, Beni-Suef University, Beni-Suef, Egypt

**Keywords:** Gingivitis, Periodontitis, Galectin, GCF, Non-surgical periodontal treatment

## Abstract

**Objectives:**

This study aimed to explore the effect of nonsurgical periodontal treatment on Galectin-1 and -3 GCF levels in gingivitis and periodontitis stage III compared to periodontally healthy individuals, to determine whether they could serve as diagnostic markers / therapeutic targets for periodontitis and revealing their possible role in periodontal disease.

**Materials and methods:**

Forty-five systemically healthy participants were included and equally subdivided into three groups: gingivitis, periodontitis (stage III), and a periodontally healthy control group. The clinical parameters were recorded. Galectin-1 and -3 GCF levels were evaluated (before and after non-surgical treatment for periodontitis) using an enzyme linked immune-sorbent assay (ELISA) kit. Receiver operating characteristic (ROC) curve was performed to reveal sensitivity, specificity, predictive value, and diagnostic accuracy of both markers.

**Results:**

The study showed statistical significance between different groups regarding Galectin-3 with higher values in periodontitis and the lowest values in healthy control. Also, Galectin-1 was significantly higher in the periodontitis/gingivitis groups than in the control group. Moreover, non-surgical periodontal treatment in periodontitis patients caused a statistical reduction in clinical parameters and biomarkers. ROC analysis revealed excellent diagnostic ability of both biomarkers in discriminating periodontitis/gingivitis against healthy individuals (100% diagnostic accuracy for Galectin-1 and 93% for Galectin-3, AUC > 0.9) and acceptable diagnostic ability between periodontitis participants against gingivitis (73% diagnostic accuracy for Gal-1 and 80% for Gal-3, AUC > 0.7).

**Conclusions:**

Both Galectin-1 and Galectin-3 seem to have outstanding diagnostic accuracy for the identification of periodontal disease, an acceptable ability to measure periodontal disease activity and the severity of inflammatory status. Additionally, they could serve as therapeutic targets to monitor treatment efficiency.

**Clinicaltrial.gov registration number:**

(NCT06038812).

## Introduction

Periodontitis (PD) lies under the umbrella of disorders having a chronic inflammatory multifactorial nature resulting from a dynamic interaction between dental plaque pathogenic bacteria, host immunologic response, as well as environmental factors, and is characterized by progressive alveolar bone and periodontal ligament destruction [1–3].

Galectins, a group of conserved proteins having carbohydrate-recognition domains (CRD) with great β-Galactosides affinity, had obtained a raising attention as chronic inflammatory disorders and tumor therapeutic targets [4–7]. Most Galectins are intracellular proteins produced on the ribosomes then transferred to cytosol and cytoplasmic membrane. They also could be found extracellularly [8, 9].

Among these, Galectin-3 (Gal-3), a conserved lectin with controversial pro- or anti-inflammatory roles according to whether located intra- or extracellularly and to its specific target cell, is produced by numerous cells like epithelial cells, fibroblasts and immune cells and exerts an impact on immune cells’ functions [10]. Gal-3 is a fundamental element in host defense against microorganisms as it could either act as pathogen-associated molecular pattern receptor binding with microbes directly, or as damage associated molecular pattern [11, 12]. After bacterial infections, Gal-3 is released from cells and directly induces secretion and migration of inflammatory mediators from innate immune cells [12, 13]. Moreover, Gal-3 has been involved in cell adhesion and tissue fibrosis in addition to inflammatory and immune responses [14].

Another member in the Galectin family, Galectin-1 (Gal-1), is considered one of the anti-inflammatory cytokines secreted by many cells like B-, T- lymphocytes, macrophages, fibroblasts, and many others [15, 16]. Gal-1 has a principal role in various biological aspects, including cell division, migration, apoptosis, immune modulation, as well as inflammation [17, 18]. Although the role of Gal-1 in anti-inflammation and suppression of immune system during various diseases has been shown in previous studies such as orchitis [19], airway allergic inflammation [20] and rheumatoid arthritis [21]. However, its effect could be changed by inflammatory stage, cell glycosylation state, and different additional elements [22, 23].

Evidenced in literature, microbial outline together with molecular biomarkers concentration and composition of gingival crevicular fluid (GCF) varies in healthy areas of individuals having periodontal disease from healthy areas of individuals having healthy periodontium. Also, clear differences in GCF composition exists during progression of periodontal disease with specific markers that could be utilized to foresee patient/site-based future disease consequences. All in all, research postulated the potential utilization of GCF based on its composition to pinpoint subclinical changes in recruitment of inflammatory cells, tissue metabolism and remodeling of connective tissues [24].

Although Gal-1 and -3 levels in different body fluids were previously evaluated in periodontal disease, the data available in literature is very limited. Moreover, the effect of non-surgical periodontal treatment was not investigated before, regarding Gal-1, to the best of the authors knowledge. Therefore, this study will evaluate the impact of non-surgical periodontal treatment on Galectin-1 and -3 GCF levels in patients having periodontitis comparing them with periodontally healthy and gingivitis groups.

## Materials & methods

Research ethics committee of faculty of Dentistry Beni-Suef university had given the approval to the current prospective investigation (Approval number: #REC-FDBSU/03082023-1/AM). The study was retrospectively registered on clinical trial.gov with registration number (NCT06038812). Before starting any procedure, the steps and objectives of the study were clarified to all participants, and they were asked to sign written consents.

This study examined 45 consecutive systemically free participants subdivided into 3 groups: *Group I*: patients having generalized gingivitis (*n* = 15). *Group II*: patients having generalized periodontitis stage III grade B (*n* = 15). *Group III*: age and gender matched individuals with healthy periodontium (*n* = 15). The patients were enrolled from the outpatient clinic of oral medicine, diagnosis, and periodontology department between August 2023 and October 2023.

Eligibility criteria include:

1. Non-smokers from both genders who give consent.
2. Subjects not suffering from systemic diseases, pregnancy/lactation.
3. Subjects not taking contraceptive pills, antibiotics, anti-inflammatory, or immunosuppressive drugs before inclusion in the study by 6 months.
4. Patients diagnosed with only gingivitis or periodontitis stage III.
5. Periodontitis patients who did not receive any professional periodontal treatment in the last 6 months.

All participants were subjected to medical and dental history recording along with full clinical examination. Probing depth (PD) [25], clinical attachment loss (CAL) [26], gingival index (GI) [27], and plaque index (PI) [28] were registered by a single examiner using William’s periodontal probe. All these clinical parameters were assessed for each tooth at 6 sites (mesio-buccal/lingual, disto-buccal/lingual, mid-buccal/lingual), and recorded for all included participants at baseline in addition to re-assessment after 4 weeks for group II patients following non-surgical periodontal treatment to record clinical parameters and took the second GCF samples. PI was measured according to presence/absence of the supragingival biofilm by sweeping motion of the periodontal probe around surfaces of all teeth [28]. Gingival marginal bleeding was registered along with GI [27].

The control group included healthy individuals who had clinically healthy gingiva (PD ≤ 3 mm and zero CAL), no bone loss radiographically [29]. Diagnosis of gingivitis [29] was confirmed if having GI ≤ 2, BOP > 10%, zero CAL, no radiographic bone loss and ≤ 3 mm PD whereas periodontitis diagnosis followed the new classification of periodontal disease [30] and was confirmed if patients were systemically free with > 30% of the sites were registered with PD ≥ 6, CAL ≥ 5 mm with radiographic evidence of bone loss.

Full mouth supra and subgingival scaling and root planning (SRP) for all periodontitis patients was done by ultrasonic scalers (non-optic ultrasonic NSK scalers, Japan) in addition to Gracey curettes (Dentsply, United Kingdom) under local anesthesia if required. Instructions of proper oral hygiene were given to patients of group I and II together with weekly follow up visits to ensure that patients stick to the given instructions.

GCF samples at baseline from all participants (gingivitis, periodontitis & control) were gathered after supragingival plaque removal with a manual scaler then washing with water spray and finally dried along with the isolation with cotton rolls to avoid salivary contamination. Second GCF samples from group II were obtained after 4 weeks from performing scaling and root planning. Samples were obtained from single rooted teeth via using paper points from pockets with the deepest readings to collect undiluted GCF. The obtained samples were given serial numbers and stored at -80◦C till analysis. Any contaminated samples with either saliva or blood were excluded [31].

### Quantitation of human Galectin-1 and -3 in GCF

GCF was used for determination of Galectin-1 and -3 using “Enzyme-Linked Immunosorbent Assays” (ELISA) kits provided by Bioassay Technology Laboratory with Cat. No E2989Hu and Cat. No E3449Hu respectively (Zhejiang, China). The plates have been pre-coated with Human Gal-1 or GAL-3 antibodies. Gal-1 and Gal-3 present in the sample were added and bind to antibodies coated on the wells. And then biotinylated Human Gal-1 and Gal-3 antibodies were added and bind to Gal-1 and Gal-3 in the sample. Streptavidin-HRP was then added and binds to the Biotinylated Gal-1 and Gal-3 antibodies. After incubation unbound Streptavidin-HRP was washed away during a washing step. A substrate solution was then added, and color develops in proportion to the amount of Human Gal-1 and Gal-3. The reaction was terminated by addition of acidic stop solution and absorbance is measured at 450 nm.

### Sample size calculation

To validate the suitable sample size required for this study a power sample analysis was done. Sample size was calculated based on data extracted from previously published research [32]. For testing GCF Galectin-3 as the primary outcome for three groups including a health control group, the effect size is (f = 0.854). The total sample size will be 27 patients (*n* = 9 in each group) with a power of 95%. The within group standard deviation was 15.95. Sample size was increased to 12 in each group to accommodate for 20% dropout with a total of 36 patients. Sample size was calculated using G*Power 3.1.9.7.

Receiver operating characteristic (ROC) curve analysis was performed to evaluate the diagnostic value of GCF Galectin-1 and-3 levels between the included groups.

### Statistical analysis

Categorical data were presented as frequency and percentage values and were analyzed using chi-square test. Numerical data was represented as mean and standard deviation (SD) values. They were tested for normality using Shapiro-Wilk’s test. Normally distributed data (age, and probing depth, Galectin-1, and Galectin-3) were analyzed using one-way ANOVA test followed by Tukey’s post hoc test for intergroup comparisons and paired t-test for intragroup comparisons. Other data were non-parametric and were analyzed using Kruskal-Wallis’s test followed by Dunn’s post hoc test with Bonferroni correction for intergroup comparisons and signed rank test for intragroup comparisons. Diagnostic accuracy was determined using ROC curve analysis. The best cutoff values were determined based on the highest Youden index. ROC curves were compared using z-test. Cut-off points were not prespecified from previous studies and were calculated from analyzed data. The significance level was set at *p* < 0.05 within all tests. Statistical analysis was performed with R statistical analysis software version 4.3.1 for Windows [33].

## Results

The study was conducted on 45 cases (i.e. 15 cases per group). Demographic data (Table 1) showed there were 4 (26.7%) males and 11 (73.3%) females in either the periodontitis or the gingivitis groups whereas in the healthy group there were 5 (33.3%) males and 10 (66.7%) females. The mean age of the cases in the periodontitis group was (46.07 ± 6.64) years, in the gingivitis group it was (44.15 ± 3.87) years while in the healthy group it was (46.27 ± 5.26) years. There was no significant difference between tested groups regarding gender (*p* = 0.897) and age (*p* = 0.497).

**Table 1:** Intergroup comparison of demographic data

Parameter			Periodontitis	Gingivitis	Healthy	Statistic	*p*-value
**Gender**	Male	n	4	4	5	0.22	0.897
		%	26.7%	26.7%	33.3%		
	Female	n	11	11	10		
		%	73.3%	73.3%	66.7%		
**Age (years)**	Mean ± SD		46.07 ± 6.64	44.15 ± 3.87	46.27 ± 5.26	0.71	0.497

n: number, %: percentage. *P*-value: Chi-square test. Age (mean ± SD): One-way ANOVA

Results of intergroup comparisons and summary statistics for clinical parameters and both biomarkers are presented in Table (2). For (PI) and Galectin-1, results showed periodontitis and gingivitis groups to have significantly higher values than the healthy group (*p* < 0.001). While for (GI) and (PD), they showed periodontitis group to have significantly higher values than other groups (*p* < 0.05). For Galectin-3, all post hoc pairwise comparisons were statistically significant with periodontitis group having the highest value followed by gingivitis group then the healthy group which shows the lowest value (*p* < 0.001).

**Table 2:** Intergroup comparison of different clinical parameters and biomarkers

Parameter	(Mean ± SD)			Statistic	*p*-value
	Periodontitis	Gingivitis	Healthy		
**PI**	2.07 ± 0.70^A^	2.20 ± 0.77^A^	0.80 ± 0.41^B^	23.26	< 0.001*
**GI**	2.47 ± 0.64^A^	1.80 ± 0.41^B^	NA	9.03	0.003*
**PD (mm)**	8.07 ± 1.44^A^	1.44 ± 0.20^B^	1.47 ± 0.52^B^	29.72	< 0.001*
**CAL (mm)**	7.33 ± 2.29	NA	NA	NA	NA
**Galectin-1 (ng/ml)**	13.56 ± 1.72^A^	14.71 ± 1.45^A^	9.88 ± 0.73^B^	51.35	< 0.001*
**Galectin-3 (pg/ml)**	350.04 ± 46.02^A^	312.25 ± 23.77^B^	234.07 ± 33.77^C^	41.17	< 0.001*

NA: Not Applicable, Values with different superscript letters within the same horizontal row are significantly different, *significant (*p* < 0.05). PI: plaque index, GI: gingival index, PD: probing depth, CAL: clinical attachment loss. PI, GI and CAL: Kruskal-Wallis test. PD & biomarkers: One-way ANOVA

The effect of non-surgical periodontal treatment in periodontitis group on different clinical parameters and biomarkers is presented in Table (3). Results showed that there was a significant reduction in different measured parameters and biomarkers after treatment (*p* < 0.001) including Gal-1 and Gal-3.

**Table 3:** Effect of non-surgical periodontal treatment on clinical parameters and biomarkers in periodontitis group

Parameter	(Mean ± SD)		Statistic	*p*-value
	Before treatment	After treatment		
**PI**	2.07 ± 0.70	0.67 ± 0.62	120.00	< 0.001*
**GI**	2.47 ± 0.64	0.67 ± 0.49	120.00	< 0.001*
**PD (mm)**	8.07 ± 1.44	5.13 ± 1.19	19.14	< 0.001*
**CAL (mm)**	7.33 ± 2.29	5.27 ± 1.91	10.02	< 0.001*
**Galectin-1 (ng/ml)**	13.56 ± 1.72	11.96 ± 1.80	5.50	< 0.001*
**Galectin-3 (pg/ml)**	350.04 ± 46.02	283.49 ± 43.47	4.35	< 0.001*

* Significant (*p* < 0.05). PI: plaque index, GI: gingival index, PD: probing depth, CAL: clinical attachment loss. PI, GI and CAL: Signed rank test. PD and biomarkers: paired t-test

**Fig. 1:**
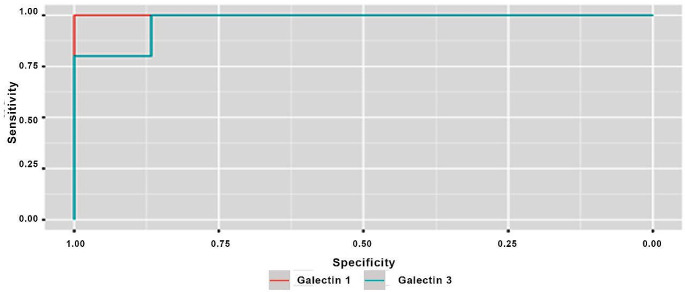
ROC curve for both markers differentiating periodontitis from healthy control

**Fig. 2:**
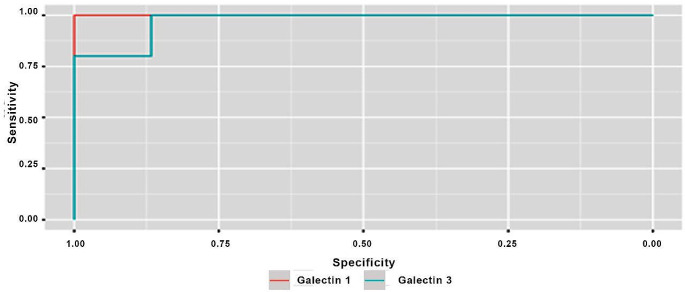
ROC curve for both markers differentiating gingivitis from healthy control

**Fig. 3:**
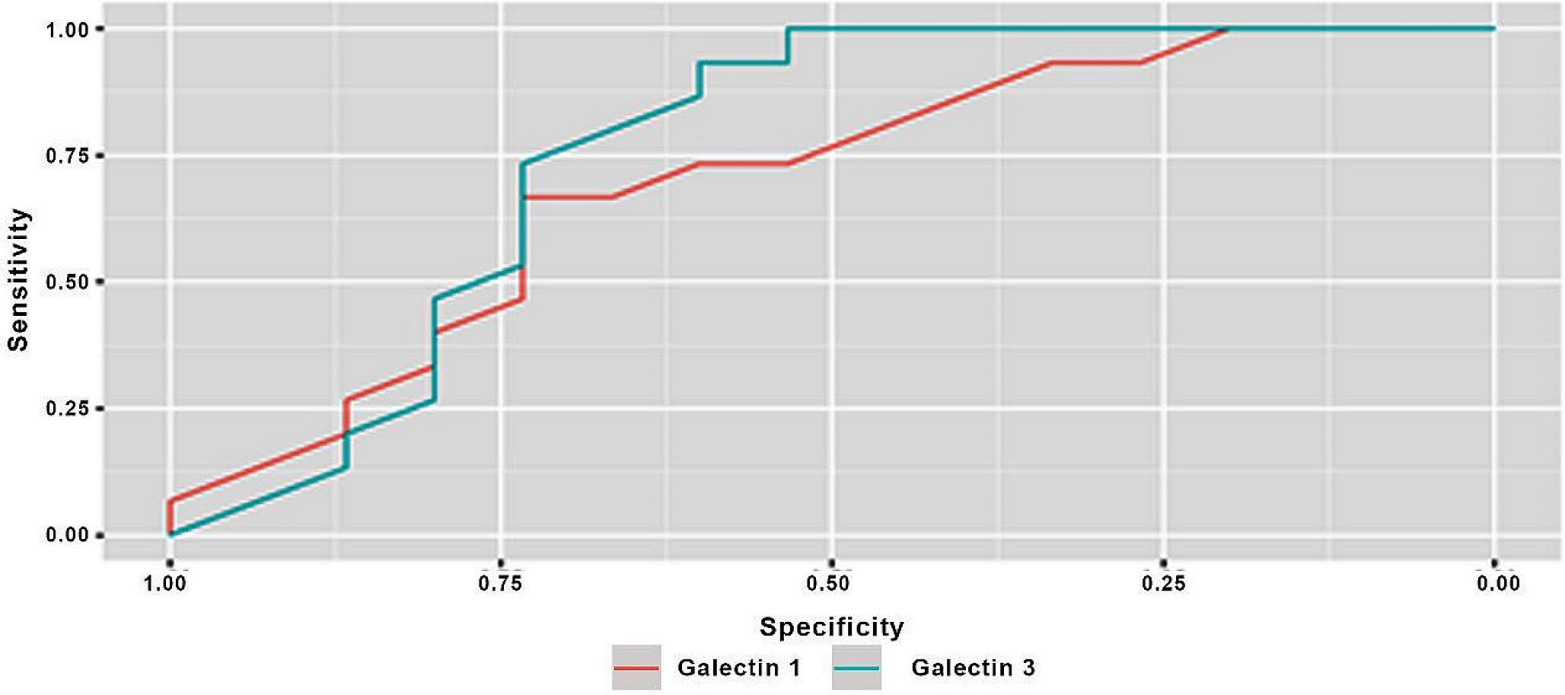
ROC curve for both markers differentiating periodontitis from gingivitis

Results of ROC curve analyses are presented in Table (4) and Figs. (1–3). Results showed both biomarkers to have remarkable diagnostic ability in discriminating periodontitis and gingivitis against healthy individuals (AUC > 0.9) and acceptable diagnostic ability in discriminating periodontitis against gingivitis (AUC > 0.7) [34] with the difference between markers being not statistically significant (*p* > 0.05). The diagnostic accuracy of Gal-1 is 100% for the differentiation between each of the diseased groups and the healthy control while Gal-3 showed slightly lower diagnostic accuracy of 93%. As for discriminating periodontitis group from gingivitis group Gal-1 showed lower diagnostic accuracy than Gal-3 with diagnostic accuracy of 73% and 80% respectively.

**Table 4:** Diagnostic accuracy of both biomarkers, cut off points, sensitivity, and specificity

Groups	Marker	Sensitivity% (95%CI)	Specificity% (95%CI)	Accuracy% (95%CI)	Cut off point	NPV% (95%CI)	PPV% (95%CI)	AUC (95%CI)	AUC difference (95%CI)	*p*-value
**Periodontitis / Healthy**	Gal-1 (ng/ml)	100.00% (100.00%:100.00%)	100.00% (100.00%:100.00%)	100.00% (100.00%:100.00%)	>=11.36	100.00% (100.00%:100.00%)	100.00% (100.00%:100.00%)	1.000 (1.000:1.000)	0.027 (-0.019:0.072)	0.284
	Gal-3 (pg/ml)	100.00% (80.00%:100.00%)	93.33% (73.33%:100.00%)	93.33% (86.67%:100.00%)	>=294.76	100.00% (83.33%:100.00%)	93.75% (78.95%:100.00%)	0.973 (0.928:1.000)		
**Gingivitis / Healthy**	Gal-1 (ng/ml)	100.00% (100.00%:100.00%)	100.00% (100.00%:100.00%)	100.00% (100.00%:100.00%)	>=11.36	100.00% (100.00%:100.00%)	100.00% (100.00%:100.00%)	1.000 (1.000:1.000)	0.027 (-0.019:0.072)	0.284
	Gal-3 (pg/ml)	100.00% (80.00%:100.00%)	93.33% (80.00%:100.00%)	93.33% (86.67%:100.00%)	>=279.21	100.00% (83.33%:100.00%)	93.75% (83.33%:100.00%)	0.973 (0.928:1.000)		
**Periodontitis / Gingivitis**	Gal-1 (ng/ml)	80.00% (46.67%:100.00%)	73.33% (26.67%:93.33%)	73.33% (60.00%:86.67%)	<=13.80	75.00% (58.33%:100.00%)	71.43% (56.52%:92.86%)	0.700 (0.507:0.893)	0.060 (-0.356:0.236)	0.691
	Gal-3 (pg/ml)	100.00% (73.33%:100.00%)	66.67% (40.00%:93.33%)	80.00% (66.67%:90.00%)	>=347.97	100.00% (75.00%:100.00%)	72.22% (60.87%:90.91%)	0.760 (0.569:0.951)		

PPV: Positive Predictive Value, NPV: Negative Predictive Value, AUC: Area Under the ROC Curve. DeLong test

Correlations between Gal-1 levels and different clinical parameters are presented in Table (5). Within different groups, all correlations were not statistically significant (*p* > 0.05). Overall, there was a strong positive correlation with PI that was statistically significant (rs = 0.651, *p* < 0.001).

**Table 5:** Correlations of clinical parameters with Galectin-1 (ng/ml)

Group	Clinical parameter	Correlation coefficient (95% CI)	*p*-value
***Periodontitis***	*PI*	-0.004 (-0.515:0.509)	0.989
	*GI*	0.275 (-0.276:0.690)	0.322
	*PD*	0.305 (-0.246:0.707)	0.269
	*CAL*	0.154 (-0.389:0.618)	0.584
***Gingivitis***	*PI*	0.311 (-0.240:0.710)	0.260
	*GI*	-0.116 (-0.593:0.421)	0.681
	*PD*	-0.293 (-0.700:0.258)	0.289
***Overall***	*PI*	0.651 (0.442:0.793)	< 0.001*
	*GI*	-0.099 (-0.444:0.271)	0.601
	*PD*	0.271 (-0.025:0.523)	0.072
	*CAL*	0.154 (-0.389:0.618)	0.584

* Significant (*p* < 0.05). PI: plaque index, GI: gingival index, PD: probing depth, CAL: clinical attachment loss. Spearman rank order correlation coefficient and z-test

Correlations between Gal-3 levels and different clinical parameters are presented in Table (6). Within different groups, all correlations were not statistically significant (*p* > 0.05). Overall, there was a moderate positive correlation with PI (rs = 0.492) and a strong positive correlation with PD (rs = 0.577) that were statistically significant (*p* < 0.001) [35].

**Table 6:** Correlations of clinical parameters with Galectin-3 (pg/ml)

Group	Clinical parameter	Correlation coefficient (95% CI)	*p*-value
***Periodontitis***	*PI*	-0.202 (-0.648:0.346)	0.469
	*GI*	-0.153 (-0.617:0.390)	0.587
	*PD*	0.269 (-0.283:0.686)	0.333
	*CAL*	0.478 (-0.045:0.796)	0.071
***Gingivitis***	*PI*	-0.058 (-0.553:0.469)	0.839
	*GI*	-0.231 (-0.665:0.319)	0.407
	*PD*	0.168 (-0.377:0.626)	0.549
***Overall***	*PI*	0.492 (0.231:0.686)	< 0.001*
	*GI*	0.189 (-0.184:0.514)	0.318
	*PD*	0.577 (0.341:0.744)	< 0.001*
	*CAL*	0.478 (-0.045:0.796)	0.071

* Significant (*p* < 0.05). PI: plaque index, GI: gingival index, PD: probing depth, CAL: clinical attachment loss. Spearman rank order correlation coefficient and z-test

## Discussion

Oral fluids being obtained easily and having local and systemic derived biochemical markers, could provide the foundation for periodontal disease specific patient diagnostic examination. Gingival crevicular fluid gathering is a non-invasive method thus it has been thoroughly investigated to discover potential diagnostic markers for periodontal diseases [24, 36, 37]. Recognizing available biochemical markers that show sensitivity/specificity and high diagnostic accuracy to prove their diagnostic and/or prognostic ability is extremely significant [38, 39]. To the best of the author’s knowledge, this is the 1st time that GCF Gal-1 and Gal-3 were investigated together in periodontal disease before and after non-surgical periodontal treatment in an attempt to validate their value to be utilized as biomarkers for periodontal disease diagnosis, showing their effect on disease progression and monitoring treatment effectiveness.

The elevated expression of serum Gal-1 and Gal-3 was related to several diseases like inflammatory/infectious diseases, tumors, and atherosclerotic stroke [40–44]. Besides chronic inflammation, Galectin-3 has been shown to play a role in acute inflammation, activated T lymphocyte proliferation, and the adhesion of neutrophils on the endothelium. Gal-3 is considered among the proinflammatory proteins which play an important role in inflammation mediated by T-cells [45]. The present study highlights this proinflammatory capacity as the herein results of GCF Gal-3 level was statistically significantly higher in periodontitis group followed by gingivitis group and finally the control group which showed the lowest value. Accordingly, we could hypothesize that Gal-3 plays a notable role in periodontal disease pathogenesis and could reflect the severity of periodontal inflammation. An investigation by Akkaya et al. [32] showed that GCF Gal-3 total amount had highest value in periodontitis group compared with gingivitis and healthy groups and the total amount in gingivitis group was also higher than the control group which was totally similar to the present results. Thus, the authors concluded that Gal-3 may have part in pathogenesis of periodontal disease owing to its elevated level in their periodontally diseased groups compared to healthy and that it could be used in periodontal disease diagnosis. Moreover, a very recent study showed similar results to ours where Gal-3 in GCF samples was found to be higher in periodontitis stage III grade B and C than gingivitis and control groups [46] which offers more evidence on our former postulation.

Earlier studies reported the therapeutic effect of Gal-3 inhibition in chronic inflammatory disorders with targeted delivery likelihood which underlines the possibility of Gal-3 being a potential therapeutic target in periodontal disease [47]. This could explain what was revealed in our results where there was statistical reduction of all clinical parameters along with Gal-3 GCF levels in periodontitis group after non-surgical periodontal treatment. Consequently, we suggest that Gal-3 could be a therapeutic target and could reflect the effectiveness of the applied treatment modality. These outcomes were supported and in accordance with a previous study [48] which also revealed similar results among their included groups where they performed initial periodontal therapy and found significant reduction in GCF Gal-3 in gingivitis and periodontitis groups compared to their baseline levels. Interestingly, the same statistical reduction was observed in our periodontitis group following periodontal treatment regarding Gal-1 GCF level which was the 1st attempt as far as we know to assess the effect of periodontal treatment on its level. Thus, it also could help in monitoring treatment efficacy.

Gal-1 has long been considered to possess anti-inflammatory effects suppressing inflammation [49] in addition it showed pro-inflammatory properties in specific circumstances allowing inflammatory damage. These double capabilities seem to be striking within neutrophils where Gal-1 could induce exposure of phosphatidylserine on activated human neutrophils encouraging activated macrophages to phagocytose them [50, 51]. In a study by Tamai et al. [52], soluble Gal-1 was found to improve invasion of P. gingivalis and its adhesion to oral epithelial cells concluding that it could promote periodontal disease progression by allowing bacterial invasion.

The present investigation revealed higher significant GCF levels of Gal-1 in periodontitis or gingivitis groups when compared to healthy group while comparing the gingivitis with the periodontitis groups insignificant higher value of Gal-1 was found in the gingivitis patients with the lowest levels existing in the healthy subjects. This could be explained by the fact that increased expression of Gal-1 in intense inflammatory conditions was an attempt to counteract this massive inflammation by acting as homeostatic mediator [53, 54]. In accordance with our results concerning Gal-1, Tasdemir et al. [55] performed a study evaluating Gal-1 level in GCF and saliva of gingivitis, periodontitis and healthy control revealed that GCF total amounts of Gal-1 was similar in gingivitis and periodontitis groups but higher than its level in control group. They concluded that elevated values of GCF Gal-1 in periodontal diseases reflect their played role in periodontal inflammation and that it could be a potential marker for periodontal disease. Moreover, they suggested that higher GCF Gal-1 values in periodontal disease groups could be attributed to extracellular matrix and collagen fibrils destruction regarding gingivitis whereas for periodontitis periodontal ligament as well as alveolar bone destruction could be the cause and that Gal-1 elevated expression was an effort to help limiting this destruction taking place in periodontal disease.

On the opposite side, a former study showed that Gal-1 upregulated profile weakened the apoptosis and autophagy in addition to production of inflammatory cytokines induced by LPS (lipopolysaccharide) in hPDLSC (human periodontal ligament stem cells) suggesting that Gal-1 could possess potential therapeutic effects on the inflammatory process of periodontal disease [56]. Several previous investigations showed that Gal-1 treatment decreases chronic inflammatory reaction and progression of diseases such as arthritis, hepatitis, and colitis [57–59]. This could also add more evidence on the concept that Gal-1 own an anti-inflammatory role in inflammatory diseases among which the periodontal disease lays.

The correlation between different markers and various clinical parameters in the present study was not statistically significant. However, overall, there is statistical significance in only PI that showed strong positive correlation with Gal-1 and moderate positive correlation with Gal-3 together with strong positive correlation with PD. This statistical insignificant correlation in each group separately could be attributed to the relatively small sample size of each group in this investigation.

ROC curve analysis showed both markers to have excellent diagnostic ability in discriminating periodontitis and gingivitis from healthy individuals (100% diagnostic accuracy for Gal-1 and 93% for Gal-3, AUC > 0.9) and acceptable diagnostic ability in discriminating periodontitis from gingivitis patients (73% diagnostic accuracy for Gal-1 and 80% for Gal-3, AUC > 0.7). Thus, they could help in diagnosis of periodontal diseases and serve as diagnostic markers.

Taken together, the results of this study concerning Gal-1 and Gal-3 GCF levels support and augment the postulation regarding their pivotal role in periodontal disease progression. In addition, based on their remarkable diagnostic accuracy for the identification of periodontal diseases and their acceptable ability to measure periodontal disease activity and severity of inflammatory status, they could serve as diagnostic markers for periodontal disease which could be clinically used as a chair side diagnostic tool. Moreover, periodontal treatment reduces their levels significantly thus they could be used to monitor treatment effectiveness.

Limitations of the current investigation include lack of contrasting Galectin GCF levels with their serum counterpart. Thus, investigations comparing GCF galectin levels with their serum levels in different stages of periodontal disease are recommended.

## Data Availability

The datasets used and/or analysed during the current study are available from the corresponding author on reasonable request.

## References

[1] Slots J (2017). Periodontitis: facts, fallacies and the future. Periodontol 2000.

[2] Papapanou PN, Sanz M, Buduneli N, Dietrich T, Feres M, Fine DH, Flemmig TF, Garcia R, Giannobile WV, Graziani F, Greenwell H, Herrera D, Kao RT, Kebschull M, Kinane DF, Kirkwood KL, Kocher T, Kornman KS, Kumar PS, Loos BG, Machtei E, Meng H, Mombelli A, Needleman I, Offenbacher S, Seymour GJ, Teles R, Tonetti MS (2018) Periodontitis: Consensus report of workgroup 2 of the 2017 World workshop on the classification of Periodontal and Peri-implant diseases and conditions. J Periodontol 89 Suppl 1S173–S182. 10.1002/JPER.17-0721 10.1002/JPER.17-0721 29926951

[3] Page, Offenbacher, Schroeder, Seymour, Kornman (1997). Advances in the pathogenesis of periodontitis: summary of developments, clinical implications and future directions. Periodontol 2000.

[4] Barondes SH, Castronovo V, Cooper DN, Cummings RD, Drickamer K, Feizi T, Gitt MA, Hirabayashi J, Hughes C, Kasai K (1994). Galectins: a family of animal beta-galactoside-binding lectins. Cell.

[5] Leffler H (2001). Galectins structure and function–a synopsis. Results Probl Cel Differ.

[6] Liu FT, Rabinovich GA (2010). Galectins: regulators of acute and chronic inflammation. Ann N Y Acad Sci.

[7] Newlaczyl AU, Yu LG (2011). Galectin-3–a jack-of-all-trades in cancer. Cancer Lett.

[8] Liu FT, Patterson RJ, Wang JL (2002). Intracellular functions of Galectins. Biochim Biophys Acta.

[9] Potikha T, Ella E, Cerliani JP, Mizrahi L, Pappo O, Rabinovich GA, Galun E, Goldenberg DS (2016). Galectin-1 is essential for efficient liver regeneration following hepatectomy. Oncotarget.

[10] Yang RY, Rabinovich GA, Liu FT (2008). Galectins: structure, function and therapeutic potential. Expert Rev Mol Med.

[11] Vasta GR (2012). Galectins as pattern recognition receptors: structure, function, and evolution. Adv Exp Med Biol.

[12] Sato S, Bhaumik P, St-Pierre G, Pelletier I (2014). Role of galectin-3 in the initial control of Leishmania infection. Crit Rev Immunol.

[13] Mishra BB, Li Q, Steichen AL, Binstock BJ, Metzger DW, Teale JM, Sharma J (2013). Galectin-3 functions as an alarmin: pathogenic role for sepsis development in murine respiratory tularemia. PLoS ONE.

[14] Loimaranta V, Hepojoki J, Laaksoaho O, Pulliainen AT (2018). Galectin-3- binding protein: a multitask glycoprotein with innate immunity functions in viral and bacterial infections. J Leukoc Biol.

[15] Villa-Verde DM, Silva-Monteiro E, Jasiulionis MG, Farias-De-Oliveira DA, Brentani RR, Savino W, Chammas R (2002). Galectin-3 modulates carbohydrate-dependent thymocyte interactions with the thymic microenvironment. Eur J Immunol.

[16] Dietz AB, Bulur PA, Knutson GJ, Matasić R, Vuk-Pavlović S (2000). Maturation of human monocyte-derived dendritic cells studied by microarray hybridization. Biochem Biophys Res Commun.

[17] Huang XT, Liu W, Zhou Y, Sun M, Yang HH, Zhang CY, Tang SY (2020). Galectin-1 ameliorates lipopolysaccharide-induced acute lung injury via AMPK-Nrf2 pathway in mice. Free Radic Biol Med.

[18] Arda-Pirincci P, Sacan O, Ozal-Coskun C, Aykol-Celik G, Karabulut-Bulan O, Yanardag R, Bolkent S (2020). Galectin-1 exhibits a protective effect against hepatotoxicity induced by dextran sulfate sodium in mice. Hum Exp Toxicol.

[19] Lei T, Moos S, Klug J, Aslani F, Bhushan S, Wahle E, Fröhlich S, Meinhardt A, Fijak M (2018). Galectin-1 enhances TNFα-induced inflammatory responses in sertoli cells through activation of MAPK signalling. Sci Rep.

[20] Lv Y, Dai M, Wang M, Chen F, Liu R (2019) Anti-inflammatory Property of Galectin-1 in a Murine Model of Allergic Airway Inflammation. J Immunol Res. 2019:9705327 10.1155/2019/9705327 PMC6535876 31214624

[21] Mendez-Huergo SP, Hockl PF, Stupirski JC, Maller SM, Morosi LG, Pinto NA, Berón AM, Musuruana JL, Nasswetter GG, Cavallasca JA, Rabinovich GA (2019). Clinical relevance of Galectin-1 and Galectin-3 in rheumatoid arthritis patients: Differential Regulation and correlation with Disease Activity. Front Immunol.

[22] Russo AJ, Vasudevan SO, Méndez-Huergo SP, Kumari P, Menoret A, Duduskar S, Wang C, Pérez Sáez JM, Fettis MM, Li C, Liu R, Wanchoo A, Chandiran K, Ruan J, Vanaja SK, Bauer M, Sponholz C, Hudalla GA, Vella AT, Zhou B, Deshmukh SD, Rabinovich GA, Rathinam VA (2021). Intracellular immune sensing promotes inflammation via gasdermin D-driven release of a lectin alarmin. Nat Immunol.

[23] Sundblad V, Morosi LG, Geffner JR, Rabinovich GA (2017). Galectin-1: a Jack-of-All-trades in the resolution of Acute and chronic inflammation. J Immunol.

[24] Barros SP, Williams R, Offenbacher S, Morelli T (2016) Gingival crevicular fluid as a source of biomarkers for periodontitis. Periodontol 2000. 70(1):53–64 10.1111/prd.12107 PMC4911175 26662482

[25] Glavind L, Löe H (1967). Errors in the clinical assessment of periodontal destruction. J Period Res.

[26] Caton J (1989) Periodontal diagnosis and diagnostic aids in proceedings of the world workshop in clinical periodontics. Chicago: the American academy of periodontology

[27] Loe H (1967). The gingival index, the plaque index and the retention index systems. J Periodontol.

[28] Silness J, Loe H (1964). Periodontal disease in pregnancy. II. Correlation between oral hygiene and periodontal condtion. Acta Odontol Scand.

[29] Chapple ILC, Mealey BL, Van Dyke TE, Bartold PM, Dommisch H, Eickholz P, Geisinger ML, Genco RJ, Glogauer M, Goldstein M, Griffin TJ, Holmstrup P, Johnson GK, Kapila Y, Lang NP, Meyle J, Murakami S, Plemons J, Romito GA, Shapira L, Tatakis DN, Teughels W, Trombelli L, Walter C, Wimmer G, Xenoudi P, Yoshie H (2018). Periodontal health and gingival diseases and conditions on an intact and a reduced periodontium: consensus report of workgroup 1 of the 2017 World workshop on the classification of periodontal and peri-implant diseases and conditions. J Clin Periodontol.

[30] Tonetti MS, Greenwell H, Kornman KS (2018). Staging and grading of periodontitis: framework and proposal of a new classification and case definition. J Periodontol.

[31] Fatima T, Khurshid Z, Rehman A, Imran E, Srivastava KC, Shrivastava D (2021). Gingival crevicular fluid (GCF): a diagnostic tool for the detection of periodontal health and diseases. Molecules.

[32] Akkaya HÜ, Yılmaz HE, Narin F, Sağlam M (2022). Evaluation of Galectin-3, peptidylarginine deiminase-4, and tumor necrosis factor-α levels in gingival crevicular fluid for periodontal health, gingivitis, and Stage III Grade C periodontitis: a pilot study. J Periodontol.

[33] R Core Team (2023) R: A language and environment for statistical computing. R Foundation for Statistical Computing, Vienna, Austria. URL https://www.R-project.org/

[34] Hosmer W Jr, Stanley Lemeshow, Rodney X, Sturdivant (2013) Applied logistic regression, vol 398. Wiley

[35] Cohen J (1988). Statistical Power Analysis for the behavioral sciences.

[36] Fine DH, Markowitz K, Fairlie K, Tischio-Bereski D, Ferrandiz J, Godboley D, Furgang D, Gunsolley J, Best A (2014). Macrophage inflammatory protein-1α shows predictive value as a risk marker for subjects and sites vulnerable to bone loss in a longitudinal model of aggressive periodontitis. PLoS ONE.

[37] Bostanci N, Ilgenli T, Emingil G, Afacan B, Han B, Toz H, Atilla G, Hughes FJ, Belibasakis GN (2007). Gingival crevicular fluid levels of RANKL and OPG in periodontal diseases: implications of their relative ratio. J Clin Periodontol.

[38] Belibasakis GN, Belstrøm D, Eick S, Gursoy UK, Johansson A, Könönen E (2023) Periodontal microbiology and microbial etiology of periodontal diseases: historical concepts and contemporary perspectives. Periodontol 2000. 10.1111/prd.12473. Epub ahead of print. PMID: 36661184 10.1111/prd.12473 36661184

[39] Gürsoy UK, Kantarci A (2022). Molecular biomarker research in periodontology: a roadmap for translation of science to clinical assay validation. J Clin Periodontol.

[40] Gauthier S, Pelletier I, Ouellet M, Vargas A, Tremblay MJ, Sato S, Barbeau B (2008). Induction of Galectin-1 expression by HTLV-I Tax and its impact on HTLV-I infectivity. Retrovirology.

[41] Saussez S, Lorfevre F, Lequeux T, Laurent G, Chantrain G, Vertongen F, Toubeau G, Decaestecker C, Kiss R (2008). The determination of the levels of circulating galectin-1 and – 3 in HNSCC patients could be used to monitor tumor progression and/or responses to therapy. Oral Oncol.

[42] ten Oever J, Giamarellos-Bourboulis EJ, van de Veerdonk FL, Stelma FF, Simon A, Janssen M, Johnson M, Pachot A, Kullberg BJ, Joosten LA, Netea MG (2013). Circulating galectin-3 in infections and non-infectious inflammatory diseases. Eur J Clin Microbiol Infect Dis.

[43] Lee PH, Liu CM, Ho TS, Tsai YC, Lin CC, Wang YF, Chen YL, Yu CK, Wang SM, Liu CC, Shiau AL, Lei HY, Chang CP (2015). Enterovirus 71 virion-associated galectin-1 facilitates viral replication and stability. PLoS ONE.

[44] He XW, Li WL, Li C, Liu P, Shen YG, Zhu M, Jin XP (2017). Serum levels of galectin-1, galectin-3, and galectin-9 are associated with large artery atherosclerotic stroke. Sci Rep.

[45] Ozaki K, Inoue K, Sato H, Iida A, Ohnishi Y, Sekine A, Sato H, Odashiro K, Nobuyoshi M, Hori M, Nakamura Y, Tanaka T (2004). Functional variation in LGALS2 confers risk of myocardial infarction and regulates lymphotoxin- a secretion in vitro. Nature.

[46] Afacan B, Ilhan HA, Köse T, Emingil G (2023). Gingival crevicular fluid Galectin-3 and interleukin-1 beta levels in stage 3 periodontitis with grade B and C. Clin Oral Investig.

[47] Velickovic M, Arsenijevic A, Acovic A, Arsenijevic D, Milovanovic J, Dimitrijevic J, Todorovic Z, Milovanovic M, Kanjevac T, Arsenijevic N (2021). Galectin-3, possible role in Pathogenesis of Periodontal diseases and potential therapeutic target. Front Pharmacol.

[48] Karsiyaka Hendek M, Olgun E, Kisa U (2021). The effect of initial periodontal treatment on gingival crevicular fluid Galectin-3 levels in participants with periodontal disease. Aust Dent J.

[49] Gil CD, Gullo CE, Oliani SM (2010). Effect of exogenous Galectin-1 on leukocyte migration: modulation of cytokine levels and adhesion molecules. Int J Clin Exp Pathol.

[50] Stowell SR, Karmakar S, Arthur CM, Ju T, Rodrigues LC, Riul TB, Dias-Baruffi M, Miner J, McEver RP, Cummings RD (2009). Galectin-1 induces reversible phosphatidylserine exposure at the plasma membrane. Mol Biol Cell.

[51] Dias-Baruffi M, Zhu H, Cho M, Karmakar S, McEver RP, Cummings RD (2003). Dimeric Galectin-1 induces surface exposure of phosphatidylserine and phagocytic recognition of leukocytes without inducing apoptosis. J Biol Chem.

[52] Tamai R, Kobayashi-Sakamoto M, Kiyoura Y (2018). Extracellular Galectin-1 enhances adhesion to and invasion of oral epithelial cells by Porphyromonas gingivalis. Can J Microbiol.

[53] Ilarregui JM, Croci DO, Bianco GA, Toscano MA, Salatino M, Vermeulen ME, Geffner JR, Rabinovich GA (2009). Tolerogenic signals delivered by dendritic cells to T cells through a galectin-1-driven immunoregulatory circuit involving interleukin 27 and interleukin 10. Nat Immunol.

[54] Fuertes MB, Molinero LL, Toscano MA, Ilarregui JM, Rubinstein N, Fainboim L, Zwirner NW, Rabinovich GA (2004). Regulated expression of galectin-1 during T-cell activation involves Lck and fyn kinases and signaling through MEK1/ERK, p38 MAP kinase and p70S6 kinase. Mol Cell Biochem.

[55] Taşdemir İ, Erbak Yılmaz H, Narin F, Sağlam M (2020). Assessment of saliva and gingival crevicular fluid soluble urokinase plasminogen activator receptor (suPAR), galectin-1, and TNF-α levels in periodontal health and disease. J Periodontal Res.

[56] Zhang J, Dong X, Yan Q, Ren W, Zhang R, Jiang X, Geng Z, Xu X, Liu C, Zhang S, Liu D, Liu Y (2021). Galectin-1 inhibited LPS-Induced Autophagy and apoptosis of Human Periodontal ligament stem cells. Inflammation.

[57] Rabinovich GA, Daly G, Dreja H (1999). Recombinant Galectin-1 and its genetic delivery suppress collagen-induced arthritis via T cell apoptosis. J Exp Med.

[58] Santucci L, Fiorucci S, Cammilleri F, Servillo G, Federici B, Morelli A (2000). Galectin-1 exerts immunomodulatory and protective effects on concanavalin a–induced hepatitis in mice. Hepatology.

[59] Santucci L, Fiorucci S, Rubinstein N (2003). Galectin-1 suppresses experimental colitis in mice. Gastroenterology.

